# Evaluation of Three Feature Dimension Reduction Techniques for Machine Learning-Based Crop Yield Prediction Models

**DOI:** 10.3390/s22176609

**Published:** 2022-09-01

**Authors:** Hoa Thi Pham, Joseph Awange, Michael Kuhn

**Affiliations:** 1 School of Earth and Planetary Sciences, Spatial Sciences Discipline, Curtin University, Perth 6102, Australia; 2 Faculty of Surveying, Mapping and Geographic Information, Hanoi University of Natural Resources and Environment, Hanoi 100000, Vietnam; 3 Geodetic Institute, Karlsruhe Institute of Technology, Engler-Strasse 7, D-76131 Karlsruhe, Germany

**Keywords:** feature selection, feature extraction, machine learning, crop yield, VCI, TCI

## Abstract

Machine learning (ML) has been widely used worldwide to develop crop yield forecasting models. However, it is still challenging to identify the most critical features from a dataset. Although either feature selection (FS) or feature extraction (FX) techniques have been employed, no research compares their performances and, more importantly, the benefits of combining both methods. Therefore, this paper proposes a framework that uses non-feature reduction (All-F) as a baseline to investigate the performance of FS, FX, and a combination of both (FSX). The case study employs the vegetation condition index (VCI)/temperature condition index (TCI) to develop 21 rice yield forecasting models for eight sub-regions in Vietnam based on ML methods, namely linear, support vector machine (SVM), decision tree (Tree), artificial neural network (ANN), and Ensemble. The results reveal that FSX takes full advantage of the FS and FX, leading FSX-based models to perform the best in 18 out of 21 models, while 2 (1) for FS-based (FX-based) models. These FXS-, FS-, and FX-based models improve All-F-based models at an average level of 21% and up to 60% in terms of RMSE. Furthermore, 21 of the best models are developed based on Ensemble (13 models), Tree (6 models), linear (1 model), and ANN (1 model). These findings highlight the significant role of FS, FX, and specially FSX coupled with a wide range of ML algorithms (especially Ensemble) for enhancing the accuracy of predicting crop yield.

## 1. Introduction

Machine learning (ML) is a branch of artificial intelligence focusing on self-learning strategies to determine the association patterns between historical yearly crop yield and yield-impacted data to provide better yield prediction. As this method outperformed biophysical models [1,2,3,4], it has been widely used recently worldwide for different crop types. However, it is still challenging when aiming to build a high-performance predictive model [2], such as selecting suitable ML algorithms or identifying the most critical features from a dataset to embellish the learning algorithm. For example, Klompenburg et al. [2] analyzed 50 studies that investigated yield prediction with different ML algorithms and concluded that models with more features did not always provide better performance for yield prediction. Therefore, models with various numbers of features should be tested to find the best-performing model. This conclusion agrees with the comment that the dimensionality reduction of components is an important area in ML [5,6], especially where datasets have many attributes [7]. It allows the ML algorithm to train faster, decreases the complexity of the model, and makes interpretation easier [7]. It also maximizes the model’s accuracy when choosing a proper subset and prevents overfitting [7].

There are two ways to reduce dimensionality: feature selection and feature extraction [5,6]. Feature selection selects only a subset of original features containing relevant information. In contrast, feature extraction transforms the input space into a lower-dimensional subspace that preserves the most pertinent information [5,6,8]. New features will not be generated in the feature selection process but through feature extraction. Although important information related to a single component is not lost in feature selection, information is likely lost as some of the features must be omitted [5]. On the other hand, in feature extraction, the feature space size can often be decreased without losing essential information about the original feature space. Still, the combination of the original components is usually not interpretable, and the knowledge about how much an initial feature contributes is often lost.

Recent studies have made much effort to apply feature selection algorithms when developing crop yield prediction models. They can be divided into three groups: (1) apply feature selection without assessing their contribution, (2) investigate which feature selection method is the best, and (3) apply feature selection and indicate its effectiveness compared with the non-feature selection approach.

Regarding the first group, various feature selection algorithms have been adopted to build a crop yield forecasting model. For example, Lingwal et al. [9] used the regularized random forest algorithm (RRF; [10]), correlation-based feature selection (CBFS; [11]), and the recursive feature elimination algorithm (RFE; [12]) to select the 10 most significant features from 18 attributes related to agriculture and weather for rice yield prediction in the Punjab State of India. Fernandes et al. [13] employed the wrapper method to exclude 14 irrelevant and/or redundant features from the initial dataset to predict sugarcane yield in São Paulo State, Brazil, based on the normalized difference vegetation index (NDVI).

Studies in the second group assessed which feature selection method is the best. For example, Gopal et al. [14] and PS et al. [8] applied five techniques, namely forward feature selection (FFS), backward feature elimination (BFE), CBFS, variance inflation factor (VIF), and random forest Variable Importance (RFVarImp), for paddy crop yield prediction in the Tamil Nadu state of India. These methods’ performance was quite similar (FFS and BFE are slightly better than others, but FFS takes less time) when combined with linear regression [8,14] and M5Prime but varied with artificial neural network (ANN) [8]. In addition, PS [7] indicated FFS performed better than CBFS, VIF, and RFVarImp. At the same time, random forest (RF) achieved the highest accuracy for all the feature subsets compared with ANN, support vector machines (SVM), K-nearest neighbor (KNN). In [15], feature selection approaches incorporated with four ML methods were investigated for bio-oil yield prediction employing biomass composition (ultimate and proximate analysis) and pyrolysis conditions (highest pyrolysis temperature, heating rate, particle size, and nitrogen flow rate). The results indicated that the genetic algorithm-based features selection approach (GA) outperforms filter and wrapper methods. Whitmire et al. [16] showed that the correlation-based method was better than ReliefF, and wrapper methods when predicting alfalfa yield in Kentucky and Georgia, the United States. Corrales et al. [17] presented the results of regression learners (linear regression (LR), SVM, backpropagation neural network (BPNN), RF, least absolute shrinkage and selection operator (LASSO), and M5 decision tree) trained with a subset of the most representative variables selected by filter, wrapper, and embedded methods to improve soybean yield prediction in Southern France. The results showed that the feature subsets selected by the wrapper method combined with SVM (6 selected features) and LR (14 selected features) provided the best results.

The third group presented the effectiveness of applying feature selection for generating a crop yield prediction model, which considers the model using all the features as a baseline. For example, Gopal et al. [14] and PS et al. [8] showed that the adjusted $R^{2}$ of 85% (84%) was achieved by using the selected features (all the features) for developing the crop yield prediction model in Tamil Nadu, India. Whitmire et al. [16] demonstrated that ML combined with feature selection offered promise in forecasting alfalfa yield even on simple datasets with a handful of features in Kentucky and Georgia. For most of the regression learners (SVM, gradient boosting regression (GBR), RF, partial least square regression (PLSR), and neuroevolution of augmenting topologies (NEAT)), using the most important parameters and the most critical months improved the coffee yield prediction in Brazil compared with employing all features [18]. Feng et al. [19] showed RF-, SVM-, and KNN-based alfalfa yield prediction in Wisconsin (the United States) were improved when using RFE-based selected features compared with those employing all features. Using satellite-derived variables, Jui et al. [20] indicated that combining RF with the dragonfly algorithm and SVM-based feature selection improved the prediction performance over other standalone ML approaches for tea yield in Bangladesh. Srivastava et al. [21,22] sorted features’ importance generated from the trained XGboost model [22] and convolutional neural network (CNN) model [21] of winter wheat yield in Germany and then developed two models based on 75 percent and 50 percent of the most important components. The models’ accuracies did not decline remarkably compared with the model based on full features; some even slightly improved. Similar results were shown in [23] for corn and soybean yield forecasting in the United States. In contrast, Bocca et al. [24] revealed that feature selection eliminated nearly 40% of the features but increased the mean absolute error (MAE) by 0.19 Mg/ha for rainfed sugarcane yield modeling in Teodoro Sampaio-São Paulo in Brazil.

These previous studies showed that feature selection had been used for determining the most important features, comparing the performance of some feature selection approaches, or the performance of feature selection for improving crop yield prediction models in different datasets. However, regarding feature extraction, the numbers of studies are fewer. For example, several studies [25,26,27,28] used principal component analysis (PCA; [29,30,31]) combined with the linear ML algorithm, i.e., the so-called principal component regression (PCR), for predicting rice yield based on vegetation condition index (VCI) and temperature condition index (TCI). However, the contribution of PCA was not transparent because they did not compare the performance of a combination of ML and PCA (PCA-ML) with ML-only. In contrast, Suryanarayana et al. [32] showed a significant improvement in PCR-based prediction models of cotton yields compared with the corresponding models using MLR. Furthermore, Pham et al. [33] proposed a new framework that considers PCA as an option for developing crop yield prediction models based on different ML methods. Using rice yield in Vietnam as a case study, the results showed that a combination of PCA with the ensemble boost tree was better than ML-only at an average of 18.5% and up to 45% of the root mean square error (RMSE) values.

A brief review of existing studies shows that either feature selection or feature extraction techniques were employed to reduce the feature dimension when developing crop yield prediction models. However, research that compares the performance of the two approaches and, more importantly, the benefits of combining both methods is still missing. Therefore, this study investigates the performance features to reduce dimensions following three options: feature selection, feature extraction, and a combination of both when developing ML modes for crop yield forecasts. The performance is measured by comparing the models generated from reduced features with those created by including all features.

This paper aims to investigate the performance of three methods of feature dimensionality reduction, namely, (i) feature selection, (ii) feature extraction, and (iii) the combination of both when developing ML-based models of crop yield prediction. It uses ML-based rice yield crop models over the entirety of Vietnam based on VCI and TCI data as a case study, which uses all common, widely used ML algorithms, namely linear, SVM, Tree, ANN, and Ensemble. Furthermore, it extends the previous work of [33] to find the best feature dimensionality reduction techniques to predict rice yield in Vietnam. To the best of the authors’ knowledge, this study provides the first comprehensive assessment of the potential of feature selection, feature extraction, and a combination of both in modeling rice yield prediction using different ML algorithms and VCI/TCI data.

Further information on reduced dimensionality methods and the main findings in the reviewed papers are shown in Table A1 of Appendix A.

## 2. Feature Selection, Extraction, and Combination within ML-Based Crop Yield Prediction Models

### 2.1. Problem Definition

Assuming that data crop yield and crop yield-influenced features are available in the same duration, ML-based crop yield prediction models can be built in the general formula as follows:

(1) $$Y\;=\;F(f_{1},\;f_{2},\ldots ,\;f_{n}),$$

where *Y* (response) denotes crop yield, e.g., rice yield; *F* refers to function (learner) of features (predictors); $f_{1}$, $f_{2}$, …, and $f_{n}$ are feature 1, feature 2, …, and feature *n*, respectively, e.g., VCI, and TCI, etc.

If a featured dataset has high dimensional data that contains many redundant or irrelevant components, it can confuse learning algorithms [33,34], making the ML-based crop yield prediction model training unstable [18], lose precision [18,35], overfit [36,37], impose a large memory space requirement [35], and a high computational cost [35,38]. Thus, feature dimension reduction has been developed to eliminate these interferences from the original feature dataset, which forms a low-dimensional feature space while maintaining as much as possible of the existing information of the data. This problem has attracted many researchers worldwide, but it is still challenging. Thus, this study is a continuation of previous achievements to propose an effective solution to this problem.

Two common ways of dimensionality reduction are feature selection (FS) and feature extraction (FX). Feature selection reduces dimensionality by selecting a small set of the original features [39]. In contrast, the FX obtains dimensionality reduction by transforming the original elements into a lower dimensional feature space [39,40]. Besides using either FS or FX, this paper proposes a combination of both techniques (hereafter referred to as FSX), as mentioned in [39]. In addition, this paper also investigates the behavior of these three feature dimension techniques: FS, FX, and FSX. Toward the abovementioned aim, a model employing all features is used as a baseline to assess models adopting FS, FX, and FSX.

**Figure 1:**
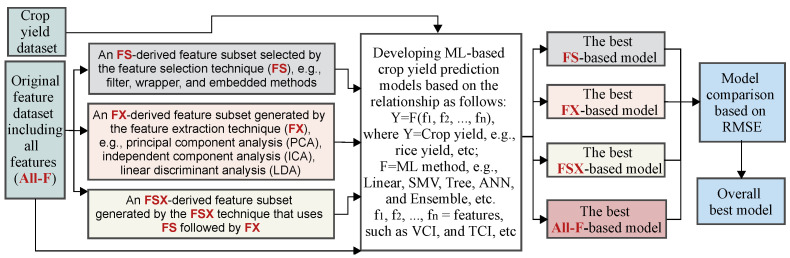
Flowchart illustrating the selection of the best performing ML-based crop yield prediction models based on FS, FX, FSX, and All-F.

The workflow of the research is designed as follows: First, crop yield prediction models are developed based on ML algorithms with (i) all the features (All-F), (ii) a feature subset obtained by using FS, (iii) a feature subset generated from FX, and (iv) a feature subset derived from FSX. Each ML method generates four FS-, FX-, FSX-, and All-F-based models using FS, FX, FSX, and All-F sets, respectively. Secondly, the best models with the highest accuracy (e.g., the model with the lowest RMSE value), namely the best All-F-, FS-, FX-, FSX-based models for feature sets derived from All-F, FS, FX, and FSX, respectively, are determined. Finally, the best FS-, FX-, and FSX-based models’ performance is compared with the best All-F-based model, and the overall best model is also determined from these four best models. The data processing is summarized in Figure 1.

Regarding the learner F in Equation (1), Klompenburg et al. [2] concluded that various ML algorithms have been used for building crop yield prediction models in previous studies, but there are no conclusions about the best model overall. This is reasonable because the crop yield forecasting models have been developed based on different feature subsets. As the “no free lunch” theory [41], models based on particular predictors for specific regions should be trained based on multiple ML methods to determine which one is the best. Thus, this work tries to use a wide range of ML algorithms such as linear, SVM, decision tree (Tree), ANN, and Ensemble. The SVM is developed with kernels as linear (SVMLine), quadratic (SMVQuaratic), cubic (SMVQuaratic), and RBF (SMVRBF). The ANN is employed with active functions such as ReLU (ANNRelu), tank (ANNTank), sigmoid (ANNSigmoid), and none (ANNNone). The Ensemble method is employed with an ensemble boost tree (EmbBoostTree), and an ensemble bagged tree (EmbBagTree). A detailed description of these ML methods is presented in [42,43].

### 2.2. Overview of Feature Selection (FS), Extraction (FX), and Combination (FSX)

#### 2.2.1. Feature Selection

Feature selection methods have been developed in the literature, which are divided into three main groups: filter, wrapper, and embedded [5,17,35,40,44,45,46]. The filter approach performs variable selection using characteristics of individual features [6]. It is uncorrelated to the training process because selecting essential features is a part of a data preprocessing step while training a model uses the selected features. The wrapper algorithm uses a specific ML algorithm based on a feature subset and then adds or removes a feature [6]. The selection criterion directly estimates the model’s change in performance caused by adding or removing a feature, determining what subsets of components lead to the best results [6,47].

Filter methods have a low computational cost and are independent of the learning method [6,48,49]. However, they lack robustness against relationships among elements and element redundancy [48,49], and it is unclear how to choose the cut-off point for rankings to determine only dominant features [6]. Generally, wrapper techniques outperform filter techniques [6,48,49] as they consider the feature dependencies and their collective contribution to model generation [6]. However, they are computationally demanding [35] and have higher risks of overfitting [6].

Embedded methods, which use the benefits of both filter and wrapper approaches, have been recently developed (e.g., [17,44,45]), which discover feature importance in the training process and are usually specific to certain learning machines [40,44]. Thus, they reduce the computational cost and improve the efficiency during the stage of FS [40].

#### 2.2.2. Feature Extraction

The most prevalent feature extraction methods are PCA [5,29,30,50], but many alternatives have been proposed recently, such as independent component analysis (ICA; [5,6,51]) and linear discriminant analysis (LDA; [6]), amongst others.

The PCA reduces dimensionality by transforming original correlated features into linearly uncorrelated components [5,29,30,31]. It computes the covariance matrix and its eigenvectors (principal components) [52]. Principle components (PCs) are new features with two properties: (1) each PC is a linear combination of the input features; (2) the PCs are uncorrelated to each other, and also, the redundant features are removed [40]. The PCs can be ranked based on the amount of variability in the data they account for. The first PCs presenting most of the variability are selected, while others are eliminated [6,30,39,40,52]. It minimizes the redundancy (estimated via the covariance) and maximizes information (estimated via the variance) [40].

The ICA is a linear transformation in which the new feature space is one that includes statistically independent components [5,6,51]. The LDA reduces the dimension by optimally projecting the initial sample to the best discriminant vector space [6,39]. The data samples after the projection have the largest inter-class distance and the smallest intra-class distance (maximum inter-class scatter matrix and smallest intra-class scatter matrix). When the original feature dimensionality is more than the number of samples, which is known as the singularity problem, LDA is not a reasonable method [40] due to the challenge of inversing the singular matrix.

#### 2.2.3. Feature Selection Combined with Feature Extraction (FSX)

Feature selection and feature extraction are currently only explored independently for developing ML-based crop yield prediction models. This paper proposes a two-step approach to combining FS and FX to reduce the dimension rather than a single step. The FS is first applied to the original feature set to select important features, followed by using FX to transform this reduced feature set obtained from the first step to lower its size further. In the first step, the FS eliminates the redundant and irrelevant features, while in the second step, the FX combines the selected elements and generates a smaller set of new features.

## 3. Case Study of Vietnam’s Rice Yield Prediction

### 3.1. Study Area

**Figure 2:**
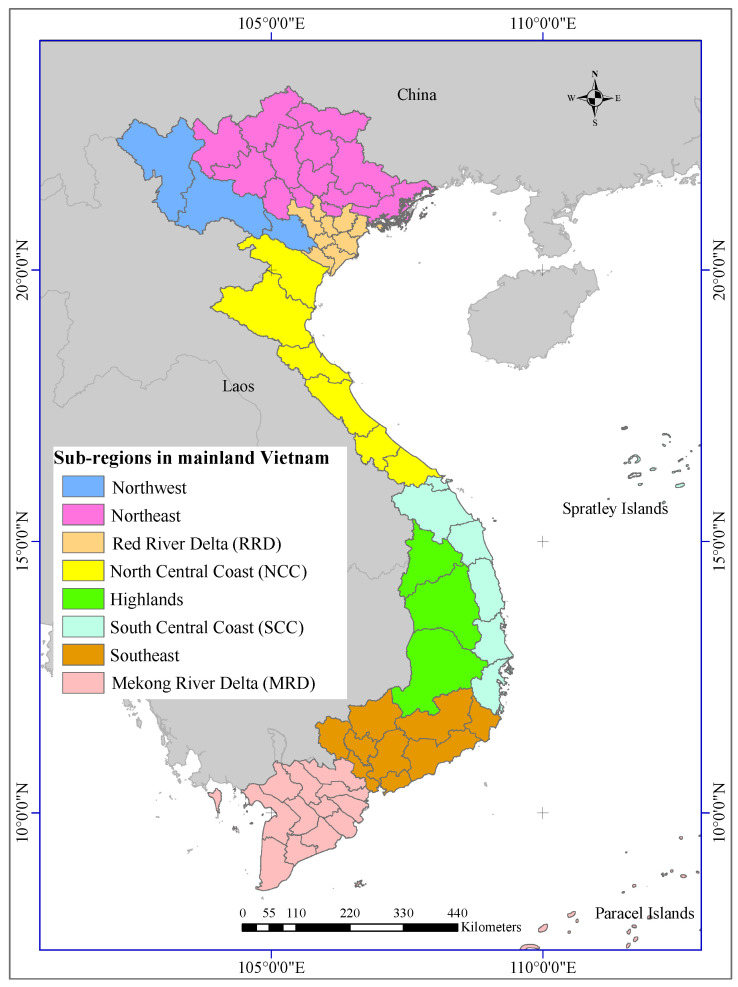
Eight sub-regions for developing rice yield prediction in mainland Vietnam: Northwest, Northeast, RRD, NCC, SCC, Highlands, Southeast, and MRD [33].

Rice is one of the three leading food crops globally [53], and Vietnam is the second-largest rice producer globally [54]. Therefore, there is a need to find out the best solution for generating accurate rice yield prediction models in Vietnam for food security. For this purpose, Pham et al. [33] divided the Vietnam mainland into eight sub-regions with uniform spatial patterns of VCI and TCI, namely Northwest, Northeast, Red River Delta (RRD), North Central Coast (NCC), South Central Coast (SCC), Highlands, Southeast, and Mekong River delta (MRD) (cf. Figure 2).

There are three rice-growing seasons in Vietnam, i.e., Winter–Spring, Fall–Winter, and Summer–Autumn. However, Northwest, Northeast, and RRD have only Winter–Spring and Fall–Winter seasons.

### 3.2. Data

#### 3.2.1. Annual Rice Yield in Vietnam

This study uses rice yield averages over all provinces in Vietnam during the 1995–2019 period obtained from the Vietnamese General Statistics Office through the link [55], accessed on 15 January 2021. A distinct sub-region’s seasonal rice yield time series is generated as a spatial mean value for 1995 to 2019, except for the Summer–Autumn rice data in the Highlands, which is for 1997 to 2019. The duration of 1995–2019 is selected because the rice yield in Vietnam is only available in this period at the time of processing data.

#### 3.2.2. VCI/TCI Data

Although several satellite-based indices have been employed to predict crop yield, there exists no empirical proof regarding which data are the best [33]. However, among others, the VCI and TCI have a theoretical advantage: They are weather-associated and, therefore, display the accumulative weather impacts on the yearly crop yield variation around the trend [56]. If they are the predictors, the yield deviations from the trend will be the responses, which avoid many other input-defining levels of crop yield stability (e.g., ecosystems, climate, soils, and topography; [57]) and form a long-term persistent yield change (e.g., pest and disease control, fertilizers, hybridization; [56]). In addition, their role as predictors has been demonstrated in crop yield forecasts at different locations [25,56,57,58]. Thus, this study utilizes VCI/TCI as predictors for developing ML-based rice yield prediction models.

The VCI, a representative of the chlorophyll and wetness of the vegetation canopy, indicates plant greenness. In contrast, the TCI characterizes thermal state [25,59] and moisture availability via radiation close to the surface and aerodynamic shapes [60]. The VCI and TCI are calculated by eradicating the long-term elements associated with climate from the NDVI and brightness temperature (BT) [61,62], respectively, so they are considered the NDVI and BT’s yearly weather-related oscillations from their climatologies. Weather changes often dominate the annual crop yield variation from a long-term yield trend [56]. Therefore, it is intensely related to the VCI/TCI, leading the VCI/TCI as predictors in producing crop yield prediction. Formulas computing the VCI/TCI from the NDVI/BT and further details related to the VCI/TCI are documented in [61].

The National Oceanic and Atmospheric Administration (NOAA) already provides the average VCI/TCI time series for eight sub-regions mentioned in Section 3.1 through the link [63], accessed on 15 December 2020. Because the rice harvested in 1995 was planted in 1994, the VCI/TCI data are selected from 1994 to 2019 to match the rice production data from 1995 to 2019. Each crop year, the average VCI/TCI comprises 52 weekly values. Missing data on weeks 37–52 in 1994, weeks 2–29 in 2004, and weeks 1–6 in 1995 are refilled by the long-term mean values of the corresponding weeks from the remaining years. The VCI/TCI data for each rice season are grouped, matching the planted time that starts after the previous harvest season and ends in the last week of the current harvest season.

### 3.3. Results

In each rice season in a particular region, the predictor and response datasets are divided into a training subset (80%) and a test subset (20%) based on a stratified sampling strategy [42] (p. 51), as performed in [33]. In all sub-regions but the Highlands, the training sets include 20-year-long predictors/responses, and the test sets include data for the left five years. Regarding the Summer–Autumn in the Highlands sub-region, the training and test sets are 19-year-long and 5-year-long time series, respectively. For further detail about preparing predictors and response datasets, see [33]. This study uses leave-one-out cross-validation for training all model, s as analyzed in [33]. The RMSE is used for model assessment. Finally, the entire data processing is implemented in the Matlab environment.

Regarding feature selection, the sub dataset is selected based on the embedded method that uses the advantages of both filters and wrapper approaches [5]. It is worth noting that embedded techniques conduct feature selection in the training process and are usually specific to certain learning machines [26,44]. However, there is a slight adaption to this study. First, embedded methods are performed by developing a model by function “TreeBagger” in Matlab to select the variables based on their importance. In this function, the feature importance is measured via the RFVarImp algorithm that uses permutation to measure how meaningful the predictors in the model are at forecasting the response. If a predictor is meaningful, permuting its values should influence the model’s performance. On the other hand, if a predictor is not effective, permuting its values will have little to no influence on the model’s performance. Finally, the features having important values above the median value (this threshold is also applied in [7]) are selected for developing FS-based crop yield prediction models. It is essential to mention here that the RFVarImp algorithm and other similar approaches were also used in recent studies to select important features when developing crop yield prediction models, e.g., [7,8,14,21,22,23].

The embedded method is a promising approach that has recently been developed to overcome the disadvantage of traditional methods such as filters and wrappers. However, relying entirely on this method is unreasonable because developing a crop yield forecasting model usually faces a small sample problem, where the feature dimensionality is high, but the number of samples is small. A small sample problem may cause most existing FS algorithms to be unreliable by choosing many irrelevant components [39] because these components can easily gain statistical relevancy due to randomness [64]. This problem can be addressed by using additional information sources to improve the understanding of the data at hand [39]. Thus, besides the embedded method, this study also uses the correlation between VCI and TCI and between VCI/TCI and rice yield to assess the collinearity among the features to select the independent ones. These outcome feature subsets are also used for developing an FS-based crop yield prediction model. Finally, these models are compared with the ones using the FS set generated from the embedded method to determine the final FS subsets for developing the FS-based model.

Regarding the FX method, this paper uses the PCA for reasons: (1) it is one of the popular FX techniques [5,29,50], and most recent studies use it when extracting features for building crop yield forecasting models, e.g., [25,26,27,28,32,33], and (2) it has confirmed records of high success in downsizing dimensions [32,33,48].

**Figure 3:**
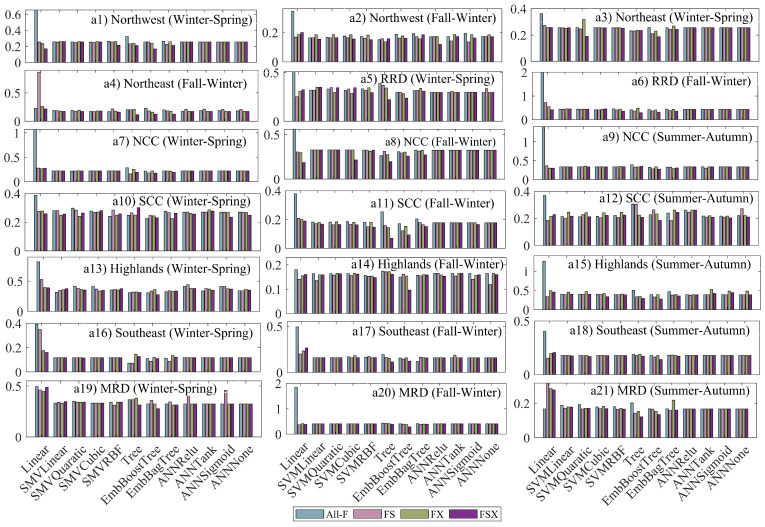
The RMSE of All-F-, FS-, FX-, and FSX-based models in different ML algorithms, units are tons/hectare.

The rice yield forecasting models are constructed based on ML approaches: linear, SVMLine, SMVQuaratic, SMVQuaratic, SMVRBF, Tree, EmbBoostTree, EmbBagTree, ANNRelu, ANNTank, ANNSigmoid, and ANNNone. For particular ML methods, four models are developed based on corresponding feature sets: All-F, FS, FX, and FSX. The RMSE of these models is shown in (see Figure 3), in which each subfigure presents a particular rice season. In a specific region, each ML algorithm includes four columns, in order from left to right, denoting the RMSE of the All-F-based model, the FS-based model, the FX-based model, and the FSX-based model. In most cases, the FS-, FX-, and FSX-based models generally perform better or at the same level as the All-F-based model except for some cases such as Winter–Spring in Northeast (Figure 3(a3)), SCC (Figure 3(a10)), and MRD (Figure 3(a19)) and Summer–Autumn in SCC (Figure 3(a12)), Highlands (Figure 3(a15)), and MRD (Figure 3(a21)). Furthermore, no clear pattern shows which model is better when comparing the FS-based and FX-based models. However, these two models offer worse performance than the FSX-based model in many cases, except in some models in Winter–Spring of SCC and MRD (see Figure 3(a10) and Figure 3(a16)) and Summer–Autumn in Highlands (see Figure 3(a15)).

The performance of the three feature subsets (FS, FX, and FSX) compared with the full feature set (All-F) varies according to ML methods and rice seasons. With specific ML, the improvement of FS-, FX-, and FSX-based models change from region to region. Within a particular sub-region, the comparison of FS-, FX-, and FSX-based model results vary depending on the ML methods used. For instance, for the Winter–Spring rice season in the Northwest, the FSX is the best if it couples with SVMRBF, Tree, EmbBoostTree, and EmbBagTree (see Figure 3(a1)). In addition, each feature subset generates varying RMSE values depending on the ML methods used. For example, in Winter–Spring of Northwest (see Figure 3(a1)), the RMSE of the FSX-based model is smaller than 0.2 ton/ha in the case of EmbBoostTree, but it is larger than 0.2 ton/ha for SVMLinear.

**Figure 4:**
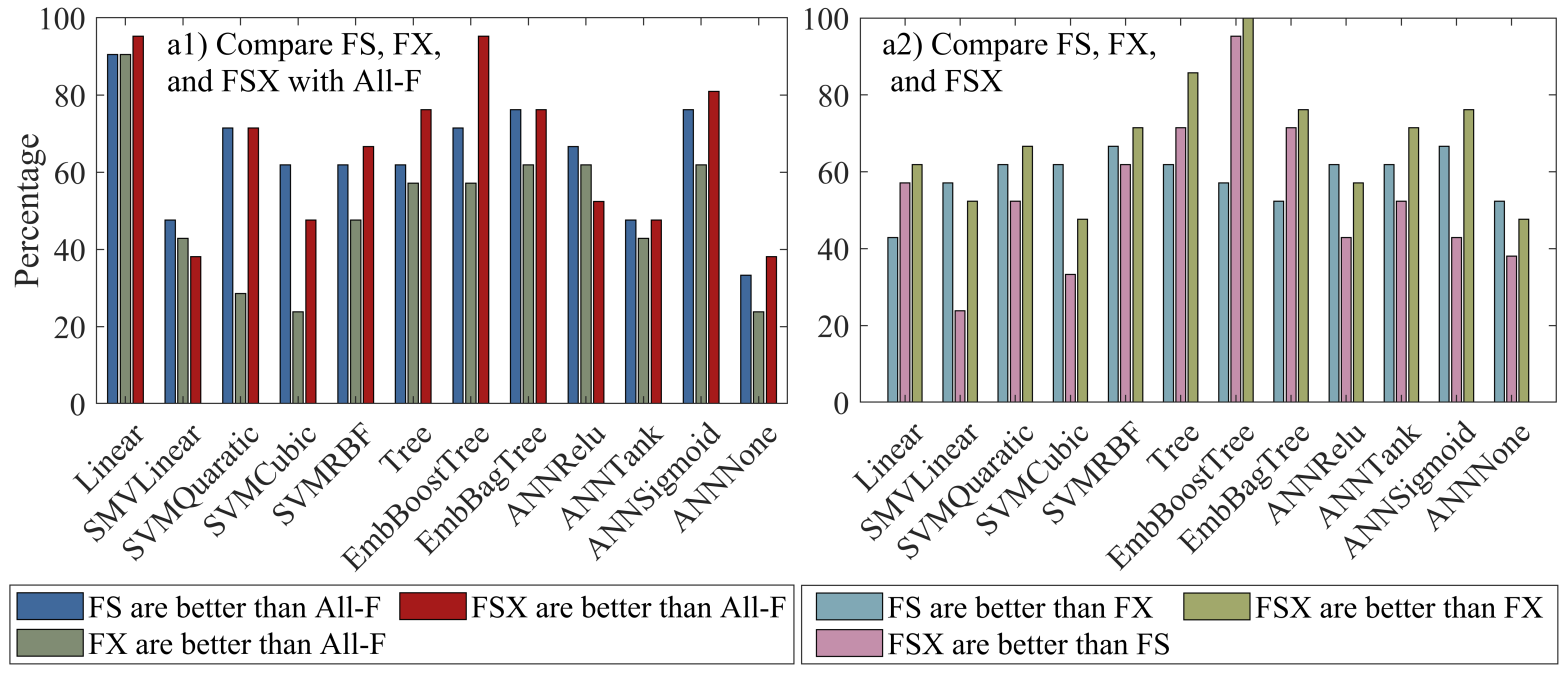
Percentage of FS-, FX-, and FSX-based models outperforming All-F-based models (**a1**); FS-based models being better than FX-based models, FSX-based models being better than FS-based models, and FSX-based models being better than FX-based models (**a2**).

The percentage of FS-, FX-, and FSX-based models outperforming All-F-based models for particular ML methods is shown in Figure 4(a1). The highest percentage for the FSX-based models (an average of 65%), followed by FS-based models (an average of 63%) and FX-based models (an average of 50%). On the other hand, the percentage of FS-based models being better than FX-based models, FSX-based models being better than FS- and FX-based models are presented in Figure 4(a2), which reveals the percentage of FSX outperforming FX (FS) is at a mean level of 68% (53%) and especially up to 100% (95%) when using EmbBoostree. The percentage of FS being better than FX varies from 42% to 67% depending on the season and has an average level of 59%.

**Figure 5:**
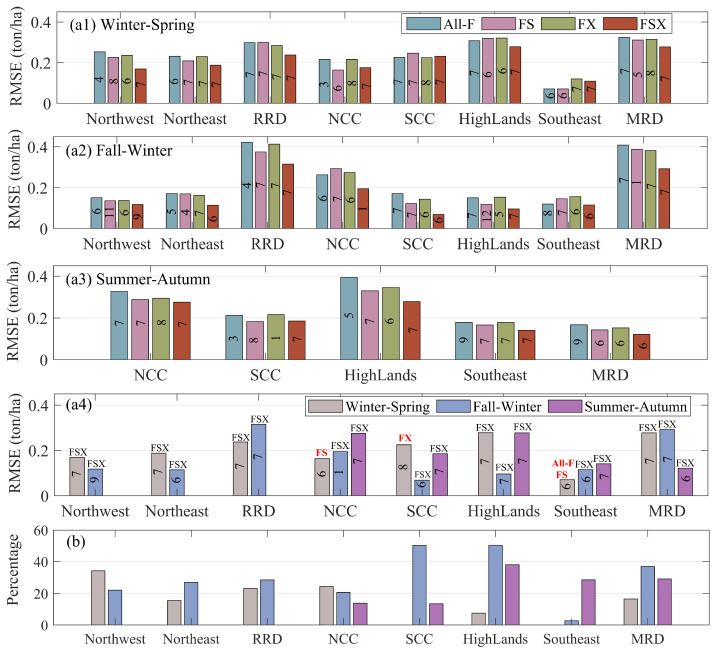
(**a1**–**a3**) display RMSE values of the best models generated from separate All-F, FS, FX, and FSX sets, while (**a4**) presents the RMSE of the overall best model selected over different feature subsets (All-F, FS, FX, and FSX) for each rice season. The numbers in the columns refer to the used ML method: 1 (linear), 3 (SVMQuaratic), 4 (SVMCubic), 5 (SVMRBF), 6 (Tree), 7 (EmbBoostTree), 8 (EmbBagTree), 9 (ANNReLu), 11 (ANNSigmoid), and 12 (ANNNone). The text at the top of the column denotes the dimensionality reduction techniques used; (**b**) The accuracy improvement of the best overall models compared with the All-F-based models.

Based on models derived from ML methods, the best model for a particular feature set (All-F, FS, FX, and FSX) is determined and presented in Figure 5(a1–a3). From left to right, the columns show the RMSE of the best model generated from All-F, FS, FX, and FSX sets, respectively. The results show that the FS, FX, and FSX sets outperform or are at least at the same level as the All-F set except for Winter–Spring in SCC and Highland (Figure 5(a1)) and Fall–Winter in NCC (Figure 5(a2)), where the RMSE of the FS- and FX-based models are larger than that of the All-F-based models. The percentage of the best FS-, FX-, and FSX-based models better than the best All-F-based models are 71%, 71%, and 90%, respectively, while 85% (90%) of the best FSX-based models are better than the best FS-based (FX-based) models. In addition, 81% of the best FS-based models are better than the best FX-based models. Those percentage values are generated from the data in Figure 5(a1–a3). Moreover, the used ML methods denoted by the number in the columns in Figure 5(a1–a3) reveal that feature subsets primarily work well with Tree (4/4/8/4 models for All-F/FS/FX/FSX), EmbBoostTree (8/10/7/14 models for All-F/FS/FX/FSX), and EmbBagTree (1/2/4 models for All-F/FS/FX).

The overall best model for each rice season (including 21 models corresponding with 21 rice seasons) is selected from the best models of all subsets (All-F, FS, FX, and FSX) and shown in Figure 5(a4). In Northwest, Northeast, and RRD, the left and right columns are denoted for Winter–Spring and Fall–Winter, respectively. For the remaining areas, the left, middle, and right columns are defined for Winter–Spring, Fall–Winter, and Summer–Autumn, respectively. The numbers in the columns display the ML methods, and the text at the top of the column stands for the dimensionality reduction techniques used. The results reveal feature sets used in the 21 best models are mainly FSX (18/21 models (86%)), followed by FS (2/21 models) and FX (1/21 model). The ML methods used in the 21 final modes include EmbBoostTree (12/21 models), Tree (6/21 models), and linear/ANNReLu/EmbBagTree (1/21 model for each).

Figure 5b indicates the improvement of the final models compared with the All-F models. The advantage ranges from 0% to 60%, fluctuating from sub-region to sub-region and from season to season. The average RMSE of these final models is smaller than that of All-F models at an average level of 0.054 tons/hectare (21%) (these data are not exhibited in Figure 5b).

## 4. Evaluation: Strengths and Limitations

Dimension reduction has become one of the most critical and challenging tasks in ML [5,6,30], which tries to obtain a valuable feature subset while maintaining the critical characteristics of the initial data [6]. Thus, in the context of using ML for developing crop yield prediction models, this paper simultaneously assessed three dimensionality reduction techniques, namely, feature selection (FS), feature extraction (FX), and the combination of the two techniques (FSX), with All-F-based model being the baseline to assess the performance of FS-, FX-, and FSX-based models. The results highlight the contribution of three feature dimension reduction techniques when developing ML-based rice yield prediction models for Vietnam based on the regression learners linear, SVM, Tree, ANN, and Ensemble using VCI/TCI data. More importantly, it revealed the superiority of the FSX over FS and FX. This main finding is further analyzed as follows:

*(1) Performance of FS, FX, and FSX for ML-based crop yield prediction models: The performance of FS, FX, and FSX compared with the baseline All-F in general:* In each ML method used, at least one of three dimension reduction methods FS, FX, and FSX performs better or at the same level as All-F except only a few cases (cf. Section 3). Specifically, considering all models, 63% of FS-based models, 53% of FX-based models, and 65% of FSX-based models outperform All-F-based models as measured by RMSE. However, regarding the best models of each feature dataset defined from all ML methods, 71%, 71%, and 90% of the best FS-, FX-, and FSX-based models, respectively, are superior to the best All-F-based models. Furthermore, the 21 final models with the highest accuracy corresponding with the 21 rice seasons, determined from the best models of all feature subsets, were developed based on FS, FX, and FSX subsets (except for one model that experiences the highest accuracy with FS and All-F). These final models improve All-F-based models at an average level of 21% and up to 60% in terms of RMSE. These findings support previous studies that concluded reducing feature dimensions is crucial for ML techniques [8,14,16,18,19,20,21,22,23,24,45]. It also pointed out that feature dimension reduction may not work well with all ML methods; thus, careful selection is necessary.

The improvement of FS, FX, and FSX also change from region to region and from season to season. This may be caused by the spatial and temporal characteristics of VCI/TCI data, the different complexity of rice yield patterns, and a limited number of samples in the case study.

*The performance of FS:* An average of 63% (71%) of FS-based models (the best FS-based models defined from all ML methods) outperform the corresponding All-F-based models. On the one hand, the results confirm the significance of the FS technique in developing crop yield prediction models, which is consistent with previous studies, e.g., for winter wheat in Germany [21,22], corn and soybean yield in the United States [23], alfalfa in the United States [16,19], rainfed sugarcane in Brazil [24], and crop in India [8,14]. On the other hand, these findings also reveal that FS is not always contributing to forecasting crop yield because the remaining FS-based models (37% for all models, 29% for the best models) do not show improvement. This outcome may be caused by the limited number of samples in the case study (20 samples for training models), which supports the previous arguments. For instance, one challenge in FS applications is the small sample issue [39]. With few samples, many irrelevant components can quickly attain statistical relevancy due to randomness [64].

The results also reveal that, except for a few cases, FS-based models are better than FX-based models while mostly worse than FSX-based models. Finally, it is worth noting that, not only in accuracy, FS also exceeds FX in providing insight into what factors most impact crop yield, leading to data collecting being more efficient [16,65].

*The performance of FX:* An average of 50% (71%) of FX-based models (the best FX-based models determined from all ML methods) are better than the corresponding All-F-based models. This finding indicates that FX does not dominate All-F all the time, highlighting the comments in [33] that PCA should only be considered as an option for developing crop yield prediction. It is important to note that the experiment in [33] was limited to the EmbBoostree method, while the present study extends to 11 other ML methods.

In addition, FX-based models are generally worse than FS- and FSX-based models. Compared with a single-level approach to reducing the dimensionality of FS, the lower quantity of FX may be explained by (1) more prominent noise existing in the VCI/TCI dataset caused by local environmental factors and (2) loss of original information due to transforming data. For the first reason, Macarof et al. [66] commented that the VCI does not perform sufficiently in wet regions. Basically, the PCA method rotates the predictors to orient the directions in which the data spread out the most with the principal axes, decreasing the data dimensionality while preserving the variance as close to the actual data as possible [30,67]. Theoretically, the PCA benefits by removing the linear correlations in the predictors leading to better results. However, when predictors contain considerable noise compared with the variance of the original data, the PCA may maximize the noise instead of the variance. Consequently, the PCA effectiveness is not always pronounced in practice [26,27,28,33,68]. The problem that PCA is sensitive to outliers in the datasets was also pointed out in [67], and this work also reviewed some techniques that referred to robust PCA as an alternative approach for simple PCA to deal with this problem. Turning to the second reason, although the FX approach tries to maintain the original behavior of the data as much as possible [39], the transformation from high-dimensional feature space to low-dimensional space will inevitably lead to the loss of some original information [6], leading to a negative effect on the resulting models. Moreover, the worse performance of FX may also come from the nonlinear relationship between predictors. Here, the FX subset is generated from PCA, which assumes the relationships between variables to be linear [5,30,67]. Thus, to overcome the limitation, the nonlinear PCA [67] should be investigated in future work.

*The performance of FSX:* The proposed FSX is expected to be superior to the single dimensionality reduction (FS or FX) techniques since it takes full advantage of both techniques. Theoretically, the FSX is better than FS because it widens FS by extracting the most important data information from the FS-based feature set. The FSX also improves the single FX technique because it reduces the dimensionality of FS-based selected features instead of the original full set of features, which far more relieves the challenge of dealing with redundant or irrelevant data. This theoretical advantage of FSX is demonstrated in the case study. An average of 65% (90%) of FSX-based models (the best FSX-based models ) perform better than the corresponding All-F-based models. Compared with single techniques, 85% (90%) of the best FSX-based models advance the best FS-based (FX-based) models. Specifically, the FSX-based models account for 86% of the 21 final models. This new finding indicates the combination (FSX) of FS and FX performs mostly better than single techniques, which extends the conclusion in [48,49] on the superiority of integrating FS and FX over single-level techniques. Here FSX is applied for predictive problems while it was employed for classification in [48,49].

*(2) The strengths of FS, FX, and FSX varies according to ML algorithms:* The results also reveal that the performance of FS, FX, and FSX varies according to ML methods. Some cases of FS, FX, and FSX being not better than All-F may be caused by inadequate training or the over-fitting phenomenon of some ML methods when using a small sample size (unfortunately, most crop yield forecasting problems worldwide face this challenge, e.g., the training set herein is 20) in the context of a highly complicated crop yield variation. This results to feature dimensionality reduction and the regression learning process becoming unstable; that is, the generalization capability of the models will drop. In addition, the 21 best models are produced by various ML methods, such as 12 EmbBoostTree-based models, 6 Tree-based models, 1 EmbBagTree-based model, 1 linear-based model, and 1 ANNReLu-based model. These results can be explained by different ways of learning the patterns of data, leading to distinct advantages and disadvantages of each ML algorithm. This outcome supports the conclusion that many ML algorithms have been used for crop yield prediction, but there is no evidence regarding which ML method is the best [2] or various ML approaches to be evaluated for specific datasets when developing models of forecasting crop yield [69]. It also underlines the “no free lunch” theory [41].

The present results of this paper also underline the ensemble learners’ potential while integrating with feature dimensionality reductions in forecasting crop yield. For example, ensemble-based models (EmbBoostTree and EmbBagTree herein) account for 13 out of 21 models (62%), while the other single learners, namely linear, Tree, and ANN, record 38%. These findings are reasonable because Ensemble is a simple and robust technique that combines the rough predictions of some weak learners to come up with accurate estimations instead of using a single ideal sophisticated learner [70]. For example, several studies demonstrated that ML ensembles could substantially outperform single ML methods [19,69,71,72,73]. Thus, there is an increasing interest in Ensemble techniques in the ML community [19]. This technique can be used in two common ways: (1) develop different training algorithms, followed by combining all the models (or several models that perform best). This approach has been used for developing prediction models (e.g., [9,19,73,74]); (2) use the same learner algorithm but train them on different subsets of the training set. It can be seen that this approach is used here by using Tree as a basic learner. Although Srivastava et al. [22] noted that there had been a marked tendency toward applying tree-based Ensemble models for developing yield prediction models recently, e.g., [69,71,72], the first Ensemble approach should also be considered in the next studies.

*(3) The limitations of this paper:* Besides the achievements mentioned above, some limitations of this paper should be noted:

*The limited numbers of techniques employed in the three feature dimension reduction:* The study has not tried the wide range of techniques for FS, FX, and FSX. Regarding FS, future work should include other methods such as filter and wrapper. Furthermore, other techniques, such as nonlinear PCA, robust PCA, ICA, and LDA, amongst others, may be investigated for FX. Again, besides the proposed FSX described in Section 2, other types of combining dimensionality reduction techniques could be considered, such as FS followed by another FS, FX followed by another FX, FS followed by another FS coupled with FX, etc.; that is, similar to some of the approaches in [48].

*Limit of predictor type:* In this case, future studies should add more predictors beyond VCI/TCI to test the performance of FS, FX, and FSX for ML-based crop yield prediction models. This is because some regions’ VCI/TCI data may not cover all short-term yield-impacted elements. For example, the VCI index represents the rainfall state, and TCI is related to thermal conditions. Thus, they represent the typical and critical factors that influence the variation of crop yields. However, in some crop areas, other factors such as irrigation regulation policy, type of seedling, and other abnormal weather variables may also affect crop yield fluctuation.

*Limit related to the small number of samples:* All 21 case studies face a small sample challenge (20 (1) case studies using 20 (19) samples for training rice yield forecasting models), leading to negative effects on the performance of reducing feature dimensionality, as mentioned above. Unfortunately, although the number of samples in this work is modest, it is still significantly more than in previous studies. In fact, long time series of most crop yield data at a regional scale worldwide is not available. Thus, it is difficult to overcome this obstacle in the near future work.

## 5. Conclusions

This paper proposed a framework for assessing different feature dimension reduction techniques, namely FS, FX, and FSX, in developing crop yield prediction models over time based on ML approaches. The experimental results underline the improvement of 21 ML-based rice yield forecasting models compared with those that do not employ feature dimension reduction. More importantly, it highlighted the superior performance of FSX (the combination of FS and FX) compared with single techniques, which have not been considered in previous works for predicting crop yield. In summary, the outcome includes four key findings as follows:

*(1) The three techniques, namely FS, FX, and FSX, improve ML-based crop yield prediction models when employing widely used ML methods to choose the best one:* 21 of the best models corresponding with 21 rice seasons, selected from all feature sets and ML methods, are all developed based on FS, FX, or FSX subsets (except for one model that could be selected from All-F- or FS-based models). These final models improve All-F-based models at an average level of 21% and up to 60% in terms of RMSE.

*(2) In general, FSX is the best technique, followed by FS and FX:* Considering all models, 65% of FSX-based models outperform All-F-based models, while this is 63% (53%) for FS-based (FX-based) models; 59% of FS-based models are better than FX-based models. With the best models selected for each feature subset: 90% (71%) of the best FSX-based (FS- and FX-based) models are better than the best All-F-based models; 85% (90%) of the best FSX-based models exceed the best FS-based (FX-based) models; 81% of the best FS-based models are better than the best FX-based models. Regarding the 21 final models selected from all feature sets and ML methods, FSX-based models account for 86%. The better performance of FSX than FS and FX may result from the accumulative advantage in both techniques.

*(3) It is necessary to consider a wide range of ML methods when applying feature dimensionality reduction techniques:* The performance of FS, FX, and FSX are not always better than All-F but depend on ML methods. The 21 final models are developed using different ML methods, including 12 EmbBoostTree-based models, 6 Tree-based models, 1 EmbBagTree-based model, 1 linear-based model, and 1 ANNReLu-based model. This finding stresses the “no free lunch” theory [41] when developing ML-based crop yield forecasting models.

*(4) Ensemble-based models have the greatest potential when combined with feature dimensionality reductions:* EmbBoostTree (12 models) and EmbBagTree (1 model) account for 13 out of 21 models (62%), while the other single learners, namely linear, Tree, and ANN, make up 38%.

This work may be helpful to other studies because it describes a framework for evaluating the three feature dimensionality reductions that can be applied to other problems related to crop yield forecasts. However, upcoming work should deal with the limitations associated with the number of techniques employed in the three feature dimension reduction by further investigating other methods, e.g., filter and wrapper for FS; nonlinear PCA, robust PCA, ICA, LDA, and so on for FX. Furthermore, besides the proposed FSX herein, other types of combining dimensionality reduction techniques for bi-level or multi-level approaches could be considered. Finally, the proposed framework should be applied with predictors beyond VCI/TCI.

## Data Availability

Not applicable.

## Appendix A

**Table A1:** The feature dimensionality reduction methods used and the main findings in reviewed papers.

Crop; Regions	Features	Methods	Related Findings
Rice; Punjab State of India [9]	Features related to agriculture and weather	RRF; CBFS, RFE	Selected ten of the most significant features
Sugarcane; São Paulo State, Brazil [13]	NDVI: At the start, in the middle, 1–10 months after the harvest starts; amplitude; max; derivative; integral	Wrapper combining ANN	Selected seven essential features
Bio-oil; Unclear [15]	Biomass composition and pyrolysis conditions	GA, filter, wrapper	GA outperforms filter and wrapper methods.
Unclear crop; Tamil Nadu, India [8,14]	Canal length; the number of tanks, tube and open wells; planting area; amount of fertilizers, seed quantity; cumulative rainfall and radiation; max/average/min temperatures	FFS, BFE, CBFS, RFVarImp, VIF	Methods were quite the same accuracy (FFS and BFE are slightly better than others, but FFS takes less time) when combined with MLR and M5Prime but varied with ANN; The adjusted $R^{2}$ of 85% (84%) was achieved by using selected features (all features).
Unclear crop; Tamil Nadu, India [7]	Canal length; the number of tanks, tube and open wells; planting area; amount of fertilizers, seed quantity; cumulative rainfall and radiation; max/average/min temperatures	FFS, CBFS, VIF, RFVarImp	FFS gives good accuracy; RF achieves the highest quality for all feature subsets compared with ANN, SVM, and KNN.
Soybean; Southern France [17]	Features related to climate, soil, and management	Filter, wrapper, embedded	The subsets selected by wrapper combined with SVM and LR provided the best results.
Winter wheat; Germany [21,22]	Weekly weather data, soil conditions, and crop phenology variables	SHAP explanation	The accuracies of models using 50/75 percent of components did not decline significantly compared with the model using full features; some even slightly improved.
Corn, and Soybean; The United States [23]	Weather components, soil conditions, and management	The trained CNN-RNN model	The models’ accuracies did not decline remarkably compared to the model based on full features, but some even slightly improved.
Alfalfa, Wisconsin, the United States [19]	Vegetation indices	RFE	All models based on RF, SVM, and KNN were improved when using selected features.
Tee; Bangladesh [20]	Satellite-derived hydro-meteorological variables	Dragonfly and SVM	Combining RF with the dragonfly algorithm and SVR-based feature selection improves prediction performance.
Alfalfa; Kentucky and Georgia [16]	Weather, historical yield, sown date	CBFS, ReliefF, wrapper	CBFS was better than ReliefF and wrapper; ML combined with FS offered promise in forecasting performance.
Sugarcane; Teodoro Sampaio-São Paulo in Brazil [24]	Soil and weather	RReliefF	FS eliminated nearly 40% of the features but increased the mean absolute error (MAE) by 0.19 Mg/ha.
Coffee; Brazil [18]	Leaf area index (LAI), tree height, crown diameter, and the individual: RGB band values	Pearson, Spearman, F-test, RFE, Mutual Information	Most of the learners using the most important parameters (LAI and the crown diameter) and the most critical months improved prediction compared with employing total features.
Winter wheat, Corn; Kansas, USA [26,27]	VCI and TCI	PCA	The contribution of PCA was unclear because PCA-ML was not compared with ML-only.
Rice, Potato; Bangladesh [25,28]	VCI and TCI	PCA	The contribution of PCA was unclear because PCA-ML was not compared with ML-only.
Cotton; Unclear region [32]	Max/min temperature, relative humidity, wind speed, sunshine hours	PCA	A significant improvement in PCR-based prediction models compared with models using MLR.
Rice; Vietnam [33]	VCI and TCI	PCA	PCA coupled with EmbBoostTree was better than ML-only at an average of 18.5% and up to 45% of RMSE.
